# Using qualitative synthesis to explore heterogeneity of complex interventions

**DOI:** 10.1186/1471-2288-11-124

**Published:** 2011-08-26

**Authors:** Bridget Candy, Michael King, Louise Jones, Sandy Oliver

**Affiliations:** 1 Marie Curie Palliative Care Research Unit, UCL Mental Health Sciences Unit, University College Medical School, Charles Bell House, 67-73 Riding House Street, London W1W 7EJ, UK; 2 Michael King, Professor of Primary Care Psychiatry and Co-Director of PRIMENT Clinical Trials Unit, UCL Mental Health Sciences Unit, University College Medical School, Charles Bell House, 67-73 Riding House Street, London W1W 7EJ, UK; 3 Louise Jones, Head of Marie Curie Palliative Care Research Unit, UCL Mental Health Sciences Unit, University College Medical School, Charles Bell House, 67-73 Riding House Street, London W1W 7EJ, UK; 4 Sandy Oliver, Professor of Public Policy, EPPI-Centre, Social Science Research Unit, Institute of Education, 20 Bedford Way, London WC1H 0AL, UK

## Abstract

**Background:**

Including qualitative evidence on patients’ perspectives in systematic reviews of complex interventions may reveal reasons for variation in trial findings. This is particularly the case when the intervention is for a long-term disease, as management may rely heavily on the efforts of the patient. Inclusion though seldom happens, possibly because of methodological challenges, and when it does occur the different forms of evidence are often kept separate. To explore heterogeneity in trial findings, we tested a novel approach to integrate qualitative review evidence on patients' perspectives with evidence from a Cochrane systematic review.

**Methods:**

We used, as a framework for a matrix, evidence from a qualitative review on patients’ perspectives on helping them manage their disease. We then logged in the matrix whether the interventions identified in a Cochrane review corresponded with the patient perspectives on how to help them. We then explored correspondence.

The Cochrane review we used included 19 trials of interventions to improve adherence to therapy in HIV/AIDS patients. The qualitative review we used included 23 studies on HIV/AIDS patients' perspectives on adherence; it translated the themes identified across the studies into recommendations in how to help patients adhere.

Both reviews assessed quality. In the qualitative review they found no difference in findings between the better quality studies and the weaker ones. In the Cochrane review they were unable to explore the impact of quality in subgroup analysis because so few studies were of good quality.

**Results:**

Matrix tabulation of interventions and patients' perspectives identified a range of priorities raised by people infected with HIV-1 that were not addressed in evaluated interventions. Tabulation of the more robust trials revealed that interventions that significantly improved adherence contained more components considered important by patients than interventions where no statistically significant effect was found.

**Conclusions:**

This simple approach breaks new ground in cross tabulating qualitative evidence with the characteristics of trialled interventions. In doing so it tests the assumption that patients are more likely to adhere to interventions that match more closely with their concerns. The potential of this approach in exploring varying content and rates of success in trialled complex interventions deserves further evaluation.

## Background

Complex multi-component healthcare interventions are challenging to evaluate [1]; variation between trials in the nature of an intervention and its effect can make it difficult to derive conclusions in systematic reviews [2,3]. The Cochrane handbook for systematic reviews acknowledges that inclusion of evidence on patients' perspectives can potentially reveal reasons for trial variation in effect [4]. Such insight, which is commonly derived from qualitative research, may inform the development of more appropriate interventions. This may particularly be the case for interventions for a long-term condition, where management can rely heavily on the patient as a co-producer of health [5]. Including such evidence with evidence on effectiveness rarely occurs. This is possibly because of the considerable methodological challenges involved [6,7]. As there are so few worked examples, a researcher attempting to do so is forced to make subjective decisions that will affect the outcome of the project. These include how the qualitative evidence is collected and analysed, and how it is used with the trial evidence.

In the worked examples available the different forms of evidence are often not brought together in analysis [8]. Instead they are reported in separate papers, where any integration of their findings is reserved for the discussion section. However, combining analytically the different types of data has the potential to reveal more about the relationship between the different forms of evidence. A realist synthesis does this by using other evidence to help explain the success or failure of a complex intervention, such as the theories or mechanisms postulated by the trial's authors or that are generated from other sources including patients [9]. However this approach, described as 'a logic of enquiry' [10, p 32] rather than a method, lacks transparency, thereby departing from the more explicit and staged approaches to reduce bias, which have distinguished and helped legitimise systematic review methods [11].

We used a contrasting approach, which conforms more to the conventions of systematic reviewing. It uses the existing primary research on a topic that meets certain criteria. It is also explicit in how it assesses and analyses the evidence. The approach explores mixed data correspondence, by tabulating in a matrix the findings of difference sources of evidence. This way of mixing the data has been used by other researchers to combine two reviews; one on patient perspectives and one on effectiveness [12]. The researchers used two-by-two matrices to link the findings from qualitative studies about children's perspectives on healthy eating with trial evidence on the effectiveness of an intervention to promote healthy eating in children. Population of the matrix represented whether there was correspondence between the two sources of data. The matrices provided an explanation for how components of the intervention contributed to its effect; for instance, interventions that increased vegetable consumption the most were those that came closest to children's views by not focusing on the health benefits, or conflating fruit and vegetables.

Constructing an evidence matrix that goes beyond four categories (i.e. two-by-two matrices) is undertaken in Qualitative Comparative Analysis (QCA) [13]. It is the first stage in data analysis, which then proceeds, if sufficient data are available, by using algorithmic logic to identify differences in data correspondence relative to the outcome of interest. By including more evidence the matrices as they are used in QCA can explore more of the relationships between the data. This approach has been used in public health topics, for instance in exploring across countries socio-economic factors relating to suicide and traffic injury [14]. We are not aware that this way of combining data has been used to explore complex healthcare interventions.

In this study we took all evidence from a qualitative review of patient's perspectives on helping them manage their disease and logged in matrices the degree to which interventions identified in a complementary Cochrane review corresponded with those perspectives.

## Method

### Data sources and selection criteria

The study used data from two published systematic reviews; one a Cochrane review on the effectiveness of an intervention, the other a qualitative review on patients' perspectives. We selected the two reviews for this study based on five criteria. The first two were on the appropriateness of the topic: (1) the disease in question required long-term self-management, and (2) the reviews' research questions closely complemented each other in disease topic. The second two were on the appropriateness of the findings: (3) the effectiveness review evaluated an intervention promoting patient self-management, identified relevant trials, and the findings of the individual trials varied in size of effect, and (4) the qualitative review analysed evidence on patients' perspectives by considering how they may contribute to appropriateness of therapies. The fifth criterion was that both reviews needed to report procedures to minimise bias in their findings. Specifically for the Cochrane review we required that they only included randomised controlled trials, and the quality of the trials included was taken into account in their findings. For the qualitative review, that they assessed quality using criteria commonly included in appraisal tools, such as those included in Critical Appraisal Skills Programme for qualitative studies [15]. Such criteria could include whether measures existed to assure validity of data collection, and that data analyses were sufficiently rigorous. We also assessed the methodological quality of the qualitative and the Cochrane review. Methods and reporting standards for undertaking a review of qualitative studies are under-developed; therefore methodological quality was assessed for both reviews using the AMSTAR [16] measurement tool to assess the methodological quality of systematic reviews. This 11-item tool includes questions on whether the review processes were undertaken in duplicate, whether the characteristics of the included studies were included, and whether the scientific quality of the included studies was assessed and documented. This tool has not been specifically designed for reviews that include qualitative evidence; therefore we also followed guidelines on how to assess quality in qualitative primary research [15,17,18]. These included assessing the validity of data by triangulation, whether methods were clear on data collection and that the findings are relevant in that they can be generalised beyond settings in which they were generated.

### Complementary reviews in HIV/AIDS

From searches in the Cochrane library and in Medline a complementary pair of reviews was identified that matched our inclusion criteria [19,20]. One review combined evidence from qualitative studies on what is known from the perspectives of HIV/AIDS patients about the problems associated with adhering to highly active antiretroviral therapy (HAART) [20]. The review identified 23 eligible studies, giving a combined total sample of 916 participants. The review authors' quality assessment found half the studies were rated as poor quality but, given their findings were in most respects comparable with the findings of the other studies, they were not excluded. The review authors describe the analysis of the studies as proceeding by 'reading the publication several times during which the findings were coded inductively in thematic groups and compared'. Thirteen primary themes were identified from the 23 studies and all themes, apart from one on homelessness, were identified in at least 7 studies. In their discussion of their findings the reviewers translated the themes into recommendations for promoting adherence. To help distinguish the type of recommendations they were grouped by WHO classification of risk factors influencing adherence to long-term therapies, namely relating to therapy, disease status i.e. having HIV-1, patient factors such as motivation to take therapy, healthcare team and system, and socio-economic circumstances [21].

The Cochrane systematic review evaluated the effectiveness of support and education strategies in improving HIV/AIDS patients' adherence to HAART [19]. This review included 19 randomised controlled trials (RCTs) giving a total sample of 2,159 participants. The reviewers did not combine trial data in a meta-analysis because of heterogeneity in population, intervention, and outcome assessment. They describe the populations across the trials as heterogeneous; specifically while most evaluated the intervention in a mixed general population others focused on high-risk groups, or particular populations such as women, children or an ethnic minority group. However, likewise there were differences in the study populations evaluated in the qualitative review. Whilst, all interventions were directed at the patient, and all were support and education-based interventions intended to improve adherence to HAART, they differed in content. A few involved cognitive management strategies, and one intervention indirectly targeted adherence by aiming to reduce risky sexual behaviours. Outcome assessment differed in how adherence was measured including, for example, pharmacy refill and self-report of the number of missed doses. The Cochrane reviewers found ten interventions were effective in increasing adherence to the prescribed medication; these were delivered to individuals rather than in groups, included six out of the seven interventions provided over 12 weeks, and six out of the eight interventions that aimed to improve practical medication skills such as by using memory aids. The reviewers used eight criteria to assess the validity of the trials findings and provided reasons for selection of the criteria based on design issues specific to adherence enhancing intervention studies [22] and the Consort Statement [23]. The criteria were: details on randomisation, concealment of allocation, an objective measure of adherence, intervention and control patients receiving similar trial contact time, follow-up for six months or longer, attrition of 20% of less in both trial arms at the end of the intervention and at six months follow-up, and that the data from all participants were analysed. Overall, the trials were reported as methodologically weak. The median criteria fulfilled were two out of a possible eight; three trials fulfilled none of the criteria. There were two distinct clusters of trials; a few fulfilling at least half of the eight criteria, and the majority fulfilling none or few criteria. As the reviewers were unable to undertake meta-analysis they could not explore in sensitivity analysis the impact of trial quality on their findings.

### Data extracted from qualitative review

We first extracted from the qualitative reviews' discussion section the recommendations for promoting adherence from the qualitative review. These we listed as 21 recommendations (Appendix 1). In accordance with the authors of the qualitative review we grouped the recommendations by the WHO classification of risk factors influencing adherence to long-term therapy [21].

### Extraction and coding of data from Cochrane review

We extracted data, from the Cochrane review, on the effectiveness of the interventions. Given that the trials measured adherence in various ways we could not standardise trial findings for the purposes of comparison. Instead we extracted data on whether the intervention was found to improve adherence significantly. One trial was excluded as it did not provide a between trial group comparison of the benefit of the intervention [24].

We planned to extract descriptions of the trialled interventions from the Cochrane review but, as the review only provided limited details on their composition, we sought this information from the original published papers reporting the trials findings. This information was central to our analysis, so we set additional inclusion criteria. Firstly, that at least a paragraph in the methods section of the paper was dedicated to describing the intervention content. Two trials were subsequently excluded [25,26]. We assessed the remaining 16 trial papers' descriptions of their interventions according to guidelines from the extension of the CONSORT statement to trials of non-pharmacologic treatment [27]. Specifically, we found all trial papers provided a description of different components of the interventions, but two did not report that the intervention was tailored to the individual and so were excluded [28,29].

Review of the 14 remaining trials revealed that all interventions aimed to improve medication management skills; this involved various techniques, for example, adaptation of dose schedules according to participants' risk factors and/or provision of adherence gadgets such as timers [30-43]. Apart from one where the comparative trial arm involved additional medication monitoring [39], all the interventions were compared with usual care. For each trial we extracted, as appropriate, the details of the intervention on to a standardised data sheet listing the 21 recommendations derived from the qualitative review. We only entered data on the trialled intervention for matches identified between components and recommendations. For example, an intervention in one trial fulfilled the recommendation *'Healthcare providers should acquire and use insight into possible factors influencing each individual patient before starting treatment' *because it was described as '*In the first visit the nurse determines the specific problems that exist with respect to knowledge and understanding of HIV infection and current antiretroviral medication adherence'*. We assessed each correspondence between the trial intervention components on whether it was a full or, if appropriate to the recommendation, a partial match. For example, a partial match would be reported if for the recommendation *'Clear information should be given on side effects how to manage side effect, including those that may be unpleasant and distressing'*, the trial authors reported that side effects were discussed but did not report that they provided information on how to manage them.

Initially, one reviewer extracted the trial data. This extraction was checked independently by two additional observers, who, although not disagreeing with the matches identified, found a small number that were missed by the other. Therefore, a further independent data extraction was undertaken by a layperson masked to the findings of the trial. Each time a passage describing the intervention matched a recommendation the layperson underlined and marked it with which recommendation it matched. This transparent method of data matching allowed us to confirm that we had not missed any matches in the unmarked text.

### Matrix for configuring and comparing the data

We used the data sheet extractions to build a matrix. Each column represented a recommendation for an intervention component derived from the qualitative review, while each row represented whether an individual trial's intervention matched any of the recommendations. We marked a cell 'X' when a commonality (a correspondence) was found between a patient recommendation and an intervention component. It was marked 'x' if only a partial correspondence was identified. It was left blank when the intervention content did not correspond with a patient-based recommendation. The final cell, per trial row, indicated whether or not the intervention was found to be effective. We used the WHO classification of risk factors influencing adherence to long-term therapy [21] as a framework to group the links found between the interventions and the recommendations. See Table 1.

**Table 1:** Tabulation of the four higher quality trials

	**Recommendations (1-21) for interventions drawn from qualitative review on patient's perspectives **[20]**sub-divided by WHO type of adherence risk factor **[21]**and if Cochrane review **[19]**found trialled intervention increased adherence (Y, N)**																					
	
	General over arching (1-3)			Therapy (4-6)			HIV-1 status (7, 8)		Patient (9-14)						Healthcare (15-18)				Social circumstances (19-21)			
	
Trialled interventions	1	2	3	4	5	6	7	8	9	10	11	12	13	14	15	16	17	18	19	20	21	
Berrien 2004			**X**	**X**							**X**	**X**	**X**		**X**	**X**	**X**					Y

Pradier 2003		x		**X**		x	**X**	x			X	**X**	x	x	**X**				**X**	**X**		Y

Rathburn 2005				**X**	x						X							x				N

Rawlings 2003				x	**X**											**X**	**X**					N

'**X**' = correspondence between the patient recommendation and the content of the intervention, 'x' = the content of the intervention partially corresponded with the recommendation.

We built two matrices. Many of the trials in the Cochrane review were methodologically weak. Therefore in our first matrix we used as a cut-off to explore heterogeneity of effect the cluster of four trials that fulfilled at least half of the set quality criteria. We explored visually whether there were differences in configurational matches in the mixed review data between the two successful interventions and the two that had no effect. In the second matrix we entered the data extractions for all trials to explore how well the patient recommendations were reflected in the components of the evaluated interventions.

## Results

The two higher quality trials in which the intervention significantly improved adherence addressed a wider range of barriers and facilitators that patients felt were important to adherence than the two higher quality trials where the intervention did not improve adherence. These latter trials did not address three types of barriers and facilitators; namely, general and overarching factors, those relating to HIV-1 status, and those relating to social circumstances (Table 1). The effective interventions both matched two recommendations that the ineffective interventions did not; specifically *'To get patients to describe their own behaviour so as to pick up any risky patterns' *and *'To develop a trusting relationship with the patient'*. There were also two recommendations that the effective trials either matched or partially matched and where no match was identified in the interventions found not to improve adherence. These were: *'To enquire for each patient before starting the treatment into the possible factors influencing adherence' *and '*Regular discussion of the details of circumstances that lead to forgetting medication can reveal aspects that need attention to improve adherence e.g. capacity to organise life and activities, and to anticipate risky situations'*. Nevertheless, there was one recommendation that was matched in the ineffective interventions that was not matched in the effective interventions, namely *'Clear information should be given on how to manage side effects, including those that may be unpleasant and distressing'*.

When we brought together in the second matrix the data extraction sheets for all 14 trials, we found few trials had included recommendations relating to HIV-1 status, or for personal social circumstances (Table 2). This included, for example, the recommendations derived from the qualitative review that *'Secrecy is threatened by taking treatment, therefore the possibility of disclosure should be discussed as openness leads to higher adherence. If disclosure is not an option, the patient can be advised how to take medication in secret to avoid skipping doses' *and *'To acquire insight into a patient's social support systems, and counsel the patient on how to use such support'*.

**Table 2:** Tabulation of all trials

	**Recommendations (1-21) for interventions drawn from qualitative review on patient's perspectives **[20]**sub-divided by WHO type of adherence risk factor **[21]																				
	
	General over arching (1-3)			Therapy (4-6)			HIV-1 status (7, 8)		Patient (9-14)						Healthcare (15-18)				Social circumstances (19-21)		
	
Trialled interventions	1	2	3	4	5	6	7	8	9	10	11	12	13	14	15	16	17	18	19	20	21
	
Berrien 2004			X	X							X	X	X		X	X	X				
Dilorio 2003																x					

Fairley 2003		**X**	**X**	**X**						x						x		**X**			

Goujard 2003		x	x	**X**																	

Levy 2004		**X**		**X**												x	x	**X**			

Murphy 2002		**X**		**X**									x				x				

Pradier 2003		x		**X**		x	**X**	x			x	**X**	x	x	**X**				**X**	**X**	

Rathburn 2005				**X**	x						x							x			

Rawlings 2003				x	**X**											**X**	**X**				

Safren 2001										x			x		x	x	**X**				

Safren 2003				**X**												**X**					

Samet 2005		**X**	**X**	**X**		x		**X**		**X**	x		**X**	x						**X**	

Smith 2003				**X**	x					**X**		**X**	**X**			x			**X**		**X**

Tulda 2000				x	x	**X**				x			**X**				**X**	x			

'**X**' = correspondence between the patient recommendation and the content of the intervention,'x' = the content of the intervention partially corresponded with the recommendation.

## Discussion

We used a simple approach to explore whether qualitative review evidence can explain the findings of a systematic review of effectiveness of a complex intervention. The approach breaks new ground by comparing in a matrix data derived from a qualitative review with data from a quantitative systematic review. By tabulating these two forms of data we were able to explore how patients' views matched the various components of trialled interventions.

The authors of the Cochrane review found that interventions targeting practical medication management skills were more often linked to successful adherence outcomes than those that did not target such skills [19]. By using our approach other information about the intervention components was revealed. Firstly, successful interventions contained a broader range of components that worked on barriers and facilitators to anti-retroviral drug adherence considered important by people infected with HIV-1. This finding is perhaps of no surprise but on the other hand this has not, to our knowledge, been formally explored before. What is surprising is that not all types of priorities raised by patients had been fully evaluated in the interventions included in the most recent update of the Cochrane review.

### Limitations

In this study any conclusions drawn on the differences between evaluated interventions are tentative as none of the trials was without risk of biased findings. Furthermore, conclusions cannot be drawn on which components are essential or superfluous, or on whether the recommendations not taken up in the reviewed trials would have any impact on adherence if included in further trials. The reason for bringing quantitative trial data together with qualitative evidence on patients' views is not to demonstrate a causal link; rather it is hypothesis generating by providing possible explanations for variation between trial findings.

Incomplete reporting in trial reports poses a threat to the validity of this approach, which is dependent on the quality of information provided on the interventions. We did attempt to ask for further information with the authors where an email address was provided. The approach was abandoned, as few responded and not all who responded provided information. However, to reduce the risk of incomplete reporting we only included trials that devoted a paragraph or more in their methods section to describing the intervention and which fulfilled the extension of the CONSORT Statement to trials of non-pharmacologic treatment on reporting of intervention content [27]. It could also be argued that while trial authors may not report all aspects of an intervention in the paper publishing their findings, they are likely to report the major components. While this has not been tested, a study of 80 papers describing evaluations of complex interventions found that the most common aspect missing from descriptions of treatments was detail on intervention process as opposed to content, such as timing of treatments [44]. Another limitation in this approach is our subjective evaluation of matches between the qualitative studies and the trials. However, to reduce this potential bias the matches identified were checked several times. Although neither of the first two checks disagreed with what had been extracted in the first extraction, each identified matches that were missed. The third check was an independent data extraction by a layperson who was masked to the trial findings. The transparent method of data matching in this final check minimised the likelihood that any further matches were missed. Finally, we were reliant on the skills of the researchers of the two published reviewers we used. To reduce this limitation we only selected reviews of a high standard.

### Implications

Systematic reviews select and combine all high quality randomised trials of a given intervention. They provide the highest quality evidence on effectiveness of a treatment or service. The approach presented here aims, by providing more evidence on intervention components, to increase the potential learning from a systematic review of a complex intervention. This information could lead to the development of more appropriate interventions.

There are a growing number of approaches for combining evidence from different types of studies. Practical experience and worked examples of different approaches are limited. This paper adds to this literature. However, it is uncertain how many other health topics would be suitable for the approach demonstrated in this paper, particularly as qualitative syntheses remain relatively uncommon and methods for qualitative synthesis vary across a range of dimensions [45]. The qualitative review we used in this study used a thematic synthesis approach [46]. Specifically, it aimed to identify all available data and to assess the quality of the studies included. The analysis involved coding of the findings of primary studies and the organisation of these 'free codes' into themes. Other researchers have explored whether a meta-ethnography of qualitative studies could potentially inform a Cochrane review on the effectiveness of interventions to promote adherence to tuberculosis treatment. Meta-ethnography was not developed as a method of systematic review but rather as a way of combining individual studies in one analysis. It involves several stages; a central stage is to translate studies into one another by in-depth review of key concepts or themes across studies [47]. One group that used the findings of meta-ethnography with evidence from a Cochrane review on adherence to tuberculosis therapy reported difficulties in translating their qualitative synthesis into recommendations for interventions. They suggested that meta-ethnography is more suitable for generating models or higher order theories of behaviour or experiences [48]. We stress however, that where reviews of studies on patients' perspectives do not present their data as recommendations in relation to a therapy, then our approach would be difficult.

The potential of the approach tested here should be further explored. While the number of reviews of qualitative evidence on patients' perspectives is small, it is slowly growing. The approach could be used to explore whether combinations of intervention components matching patients' views vary with the trials' effect sizes. Statistical analysis of the matrix data may be restricted because of the limited data points that mixed review evidence could provide but further analysis could explore, in a review with more methodologically robust trials, the value of using the algorithmic logic of QCA to explore variation in trial findings. The potential of QCA in combining qualitative and qualitative evidence synthesis in health care research is recognised by review methodologists [7] and, while we were unable to explore its full potential in the study presented here, we are currently doing so using evidence from a Cochrane systematic review where there are more trials that can be combined with qualitative review evidence [49].

## Conclusions

Our approach breaks new ground by combining qualitative and quantitative data in matrices that show whether effective interventions are more likely to contain components patients believe are important than ineffective interventions. In short, it directly tests the assumption that patients are more likely to stick to interventions that contain more of their concerns. The value of this approach in exploring heterogeneity in trial content and findings deserves further evaluation. We encourage authors publishing trial findings to provide an adequate description of their intervention. We also call for more reviews of qualitative evidence on patients' perspectives and, in particular, qualitative reviews that complement existing reviews of trials.

## Competing interests

The authors declare that they have no competing interests.

## Authors' contributions

All authors contributed to the conception, design and interpretation of the data.  BC drafted the article and all authors revised it and approved the final version

## Appendix 1: Recommendations for adherence interventions drawn from the qualitative review on HIV/AIDS patient's perspectives*

### General and overarching

1. Adherence is a dynamic phenomenon in which the interplay of a number of influences varies over time. Therefore ongoing attention to adherence should have highest priority.

2. To enquire into the possible factors influencing adherence for each individual patient before starting treatment.

3. To use insight into the possible adherence factors influencing each individual patient before starting treatment.

### Therapy related

4. Medication should be adapted to life rather than life to medication: e.g. the provider should encourage the use of a watch or pillbox with an alarm to remind a patient to take medication and to prevent (unwanted) disclosure.

5. Clear information should be given on side effects how to manage side effect, including those that may be unpleasant and distressing.

6. During every visit any ambivalence towards medications should be discussed.

### Condition related

7. Patients' acceptance of their HIV status should be discussed on a regular basis, bearing in mind that taking medication can renew confrontation with diagnosis.

8. Secrecy is threatened by taking treatment. Therefore the possibility of disclosure should be discussed as openness leads to higher adherence: if disclosure is not an option, the patient can be advised how to take medication in secret to avoid skipping doses.

### Patient related

9. Feedback about positive reactions of the body to treatment should be provided at each visit to promote adherence e.g. decreased viral load.

10. Pointing out the value of treatment to a patient's life enhances motivation.

11. Information transfer appropriate to a patient's level of understanding will lead to the patient having correct knowledge of what constitutes good adherence practice.

12. It is important to get patients to describe their own behaviour so as to pick up any risky patterns.

13. Regular discussion of the circumstances that lead to forgetting medication can reveal aspects that need attention to improve adherence e.g. capacity to organise life and activities, and to anticipate risky situations.

14. Depression or substance misuse should be managed as first priority.

### Healthcare related

15. To develop a trusting relationship with the patient.

16. To give clear instructions on how to take medication.

17. To explain the relationship between adherence and viral load.

18. To offer good medical follow up.

### Socio-economic related

19. To acquire insight into a patient's social support systems, and counselled the patient on how to use such support.

20. Attention to possible negative social circumstances e.g. mothers of young children may need help to fit medication into hectic schedules.

21. Social support has to be substantial and practical.

*Derived from Vervoort review [20]*and grouped by WHO *[21]*type of adherence risk factor*

## Pre-publication history

The pre-publication history for this paper can be accessed here:

http://www.biomedcentral.com/1471-2288/11/124/prepub
